# A New Escherichia coli Entry Vector Series (pIIS18) for Seamless Gene Cloning Using Type IIS Restriction Enzymes

**DOI:** 10.1128/MRA.00323-19

**Published:** 2019-10-10

**Authors:** Hend Altaib, Yuka Ozaki, Tomoya Kozakai, Yassien Badr, Izumi Nomura, Tohru Suzuki

**Affiliations:** a The United Graduate School of Agricultural Science, Gifu University, Gifu, Japan; b Faculty of Applied Biological Sciences, Gifu University, Gifu, Japan; c Department of Animal Medicine, Faculty of Veterinary Medicine, Damanhour University, El-Beheira, Egypt; University of Maryland School of Medicine

## Abstract

A series of new Escherichia coli entry vectors (pIIS18-SapI, pIIS18-BsmBI, pIIS18-BsaI, pIIS18-BfuAI-1, and pIIS18-BfuAI-2) was constructed based on a modified pUC18 backbone, which carried newly designed multiple cloning sites, consisting of two facing type IIS enzyme cleavage sites and one blunt-end enzyme cleavage site. These vectors are useful for seamless gene cloning.

## ANNOUNCEMENT

The plasmid construction in Escherichia coli is one of the essential routine works in the field of molecular biology (1, 2). The seamless (or scarless) gene cloning technique is an important tool for precise assembly of DNA fragments which leaves no additional linker sequence between assembled fragments. This method enables the creation of an ideal condition for precise functional studies, such as mutation study, gene fusion, and genome engineering (3, 4). Recently, the seamless cloning techniques, such as Golden Gate cloning (GGC) (5, 6) and Gibson assembly (7), have been developed and widely used in various genetic engineering applications.

**FIG 1:**
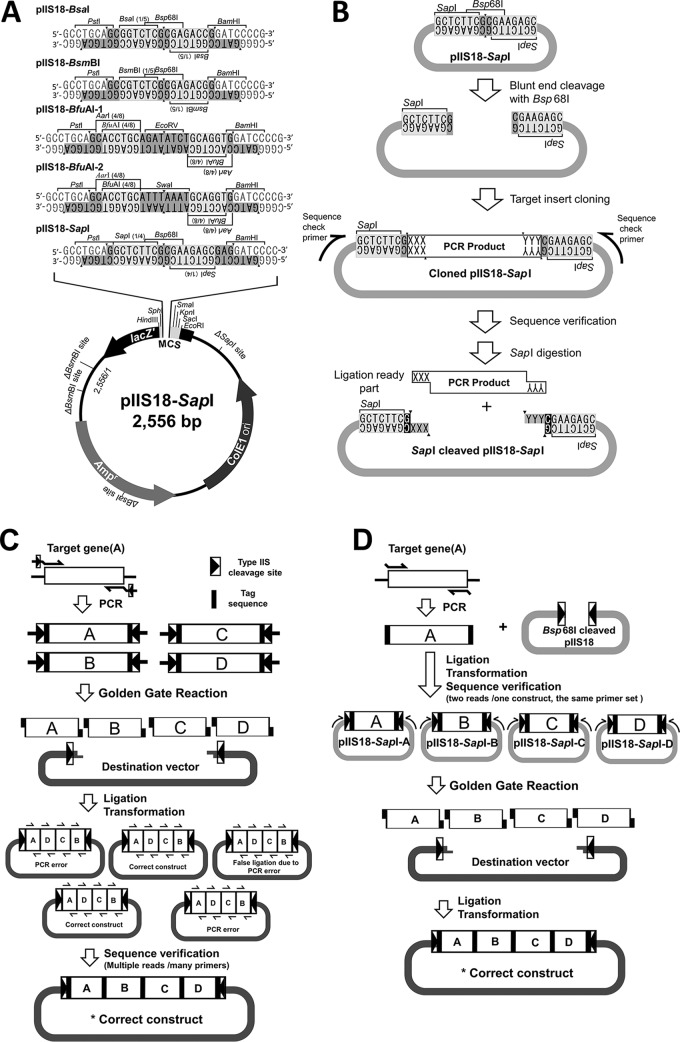
(A) Molecular structure of pIIS18-SapI cloning vector series, showing genes on the plasmid backbone. The removed type IIS enzyme cleavage sites from pUC18 are marked with parentheses on the plasmid map. The MCS structure is illustrated within the *lac*Z′ gene. The DNA linkers inserted into the MCS of each plasmid construct are demonstrated. Each linker carries one blunt-end enzyme and two facing type IIS enzyme cleavage sites located between PstI and BamHI sites. (B) Model for usage of the pIIS18 entry vector, demonstrating the insertion of a 3-bp tagged PCR product within the blunt-end-cleaved pIIS18-SapI. Once a fragment is inserted in pIIS18, it can be sequence verified and then become a ligation-ready part. (C) Original GGC, in which PCR errors make it possible to get a mutated construct; hence, it requires multiple proof sequence reads to find out the correct construct and requires multiple primer design. (D) GGC with pIIS18, in which PCR errors were eliminated through an additional cloning and sequencing step before GGC. The additional step removes the possibility of PCR error construct and allows the multiple usage of sequence-verified DNA parts several times. Few sequence reads will be needed just to confirm fragment order in the final construct.

Type IIS restriction enzymes recognize a 5- to 8-bp asymmetrical sequence and cleave outside the recognition sequence (8). This unique feature fits for the seamless cloning method and is used in GGC. Usually, PCR-amplified fragments are used for GGC or other seamless cloning techniques. However, each fragment needs 10 or more excess bases to be added at the 5′ end of each primer (Fig. 1C), which may disturb PCR amplification. Using a PCR fragment, it also needs to confirm the DNA sequence to obtain a correct clone because the DNA polymerases do not have 100% fidelity.

Several expression vectors have become available for GGC and other seamless cloning techniques. In this paper, we focused on constructing a new series of entry vectors, pIIS18-SapI, pIIS18-BsmBI, pIIS18-BsaI, pIIS18-BfuAI-1, and pIIS18-BfuAI-2. Each vector carries a newly designed multiple cloning site (MCS) on a modified pUC18 backbone (1, 9). We constructed this plasmid series as described below.

Five different series of DNA linkers were designed to include two facing type IIS enzyme cleavage sites (SapI, BsmBI, BsaI, or BfuAI) and one blunt-end enzyme cleavage site (EcoRV, Bsp68I, or SwaI) (Fig. 1A). Each design retains the same reading frame of the β-galactosidase gene (*lacZ*′). pUC18 carries two BsmBI sites, one SapI site, and one BsaI site within its backbone. We have removed these sites using site-directed mutagenesis. This will prevent jamming (incorrect) religation in case of the one-pot reaction of GGC (Fig. 1D) (5).

For pIIS18-SapI construction, pUC18 was doubly digested with PstI and BamHI. The linearized pUC18 was purified and ligated with the SapI DNA linker (Fig. 1A). The obtained ligation product was introduced to E. coli DH5α chemically competent cells (Nippon Gene), following the standard protocol, and colonies were selected on an LB agar plate supplemented with ampicillin (100 μg/ml) and 2% X-Gal (5-bromo-4-chloro-3-indolyl-β-d-galactopyranoside). The modified construct (within blue colonies) was confirmed by successful cleavage with SapI or Bsp68I and whole-plasmid sequencing using the BigDye Terminator ver. 3.1 cycle sequencing kit. The sequence data were analyzed using an ABI 3130xl genetic analyzer (Thermo Fisher Scientific, Inc.). The other four pIIS18 vector models have been constructed in the same way as pIIS18-SapI.

pIIS18 series are designed for type IIS restriction enzyme-mediated seamless gene fusion, such as GGC (Fig. 1D). They allow for direct sequence analysis of a cloned DNA fragment with just a single primer set annealing to the plasmid backbone before seamless ligation (Fig. 1B). The mutation rate during the ligation and transformation reactions is practically ignorable. The pIIS18 series will be helpful for molecular biologists, especially in experiments requiring DNA sequence verification, such as multigene fusion (Fig. 1D), and systematic construction of mutants.

### Data availability.

The complete sequences of pIIS18-SapI, pIIS18-BsmBI, pIIS18-BsaI, pIIS18-BfuAI-1, and pIIS18-BfuAI-2 have been deposited in the DNA Data Bank of Japan under the accession numbers LC459971 to LC459975, respectively. The resource can be obtained from the Addgene depository (https://www.addgene.org/) and the GCMR library of Gifu University (https://www1.gifu-u.ac.jp/~g_cmr/index.html). The raw sequencing reads are available at https://www1.gifu-u.ac.jp/~suzuki/pIIS_plasmids/.
